# Physical activity support for people with heart failure: mixed-methods study protocol

**DOI:** 10.3399/BJGPO.2025.0030

**Published:** 2025-08-27

**Authors:** Vincent Singh, John Percival, Olivia Skrobot, Rasha Okasheh, Rachel Johnson, Alyson L Huntley

**Affiliations:** 1 School of Health and Social Wellbeing, College of Health, Science and Society, University of the West of England, Bristol, UK; 2 Centre of Academic Primary Care (CAPC), Bristol Medical School, University of Bristol, Bristol, UK; 3 School of Allied Health Professions, MacKay Building, Keele University, Keele, UK

**Keywords:** heart failure, physical activity, primary healthcare, general practitioners, cardiovascular disease

## Abstract

**Background:**

Heart failure (HF) is a common condition affecting 1–2 in every hundred adults in the UK and one in six people aged over 85 years. Physical activity is important for people with heart failure (PWHF) as it can increase exercise capacity and is shown to improve quality of life. We know that PWHF often seek advice from healthcare professionals about participating in physical activity safely, but healthcare professionals are not always sure what physical activity advice is best for their patients. It is also important to understand what physical activity services are available for healthcare professionals to refer patients to and to explore how patients are referred into these physical activity services.

**Aim:**

To explore the barriers and enablers to primary and community healthcare professionals (GPs and nurses) referring PWHF to community physical activity services.

**Design & setting:**

A mixed-methods study will be conducted across GP practices in England.

**Method:**

The following approaches will be used: i) in-depth qualitative interviews with GPs (*n* = 12) and nurses (*n* = 12) to understand their experiences of talking to PWHF about referral to physical activity services; ii) finding out what commissioned physical activity services are available to PWHF, and how primary and community healthcare professionals can refer patients to the services; iii) two workshops to examine the findings with our stakeholders.

**Conclusion:**

The findings will be used to inform the development of an intervention for healthcare professionals referring to physical activity services and support the community service configuration.

## How this fits in

People with heart failure (PWHF) want to know from healthcare professionals what physical activity they can safely do but healthcare professionals are not always sure what physical activity advice is best for their patients. It is also unclear what physical activity services are available for healthcare professionals to refer their patients to. This study aims to find out what are the barriers and enablers to primary and community healthcare professionals referring PWHF to community physical activity services.

## Introduction

Nearly 1 million people in the UK are living with heart failure (HF).^1^ Common symptoms include breathlessness, fatigue, and leg swelling, which can lead people with heart failure (PWHF) to experience a diminished quality of life and repeated hospitalisation. In addition to the impact on people’s lives, it is estimated that it costs the NHS £2 billion per year to treat PWHF.^2^

Physical activity is important for PWHF as it can increase exercise capacity and is shown to improve quality of life.^3–8^ A recent study of 78 500 healthy people found that those who walked more daily steps had reduced risks of cancer, cardiovascular disease, and early death, which concurs the broader benefits of being physically active.^9^ Evidence from structured exercise-based cardiac rehabilitation shows a positive effect on mortality and hospitalisations for those who have had a heart procedure, surgery, or with a diagnosed cardiovascular disease. It is reasonable to assume that if general physical activity by PWHF is optimised it could also improve these outcomes. Most people diagnosed with HF are managed in primary care and may or may not be referred to specialists or provided with a treatment plan (including for physical activity) for their condition.^10,11^

Our previous research highlighted that PWHF want endorsement and support from community healthcare professionals to give them confidence and alleviate fears around physical activity.^12^ However, healthcare professionals lack confidence in discussing physical activity and are uncertain how and where to refer patients for physical exercise provision and/or support. Positive reinforcement by professionals is considered paramount to continued participation in physical activity for PWHF.^13^

The aims of this study are to; a) provide a detailed understanding of the barriers and facilitators for GPs and nurses in their role of talking and supporting physical activity and referring to services, and b) identify the access, design, and provision of real-world community physical activity in England for PWHF.

## Method

**Figure 1.**
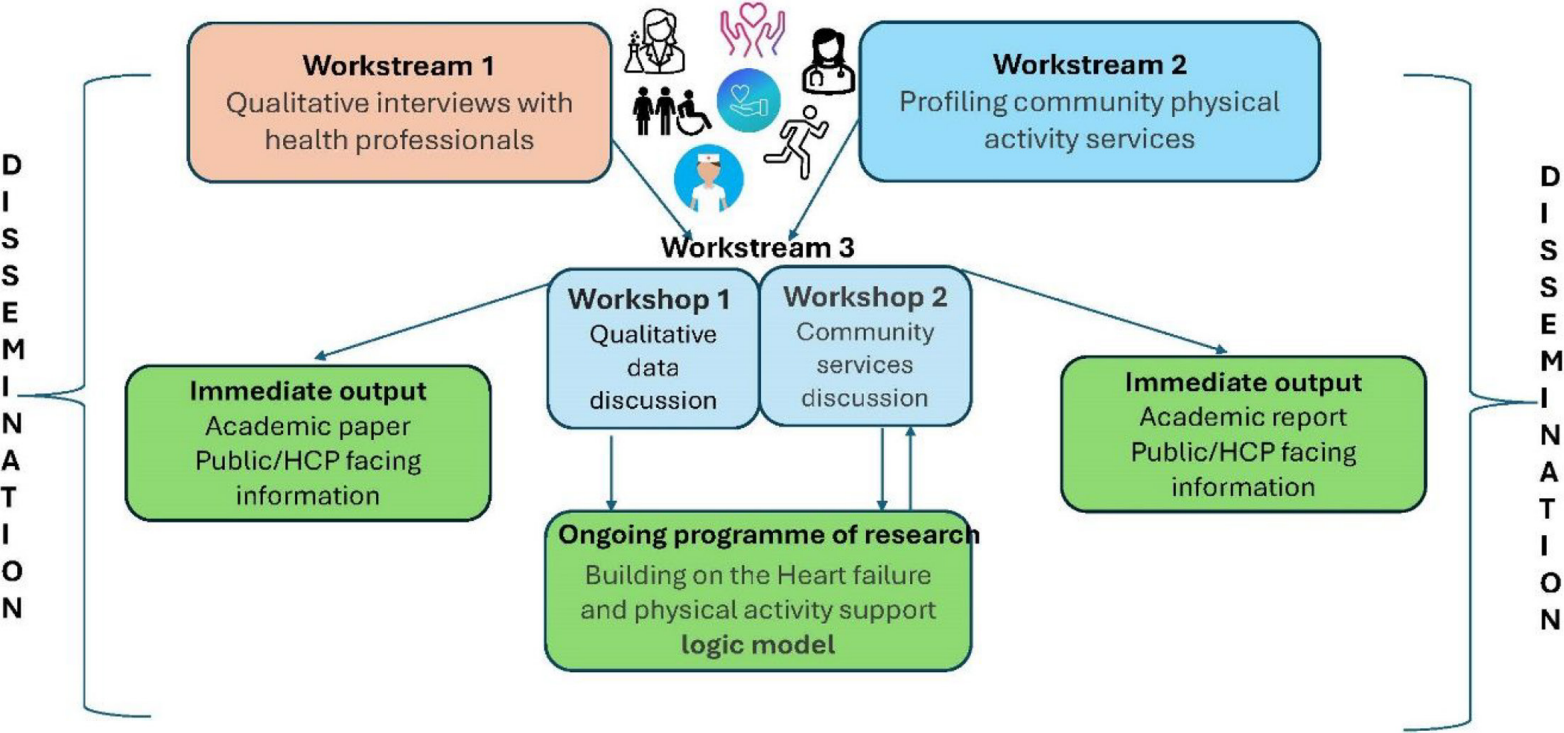
Work stream flow, outputs and dissemination

A concurrent mixed-methods approach will be followed in this study and will include qualitative and quantitative methods with stakeholder engagement (Figure 1).

### Qualitative study

#### Aim, design, and setting

The aim of this study is to understand the experiences of healthcare professionals talking to PWHF about physical activity and referring them to community physical activity services. It will be a qualitative interview study with healthcare professionals (GPs and nurses) who see PWHF as part of their role, set in GP practices in England.

#### Sampling

The qualitative study will recruit approximately 12 GPs and 12 nurses from GP practices across England. The exact sample size will be determined when saturation of the data is established, according to our key research questions and analytic framework, and will be verified by the research data analysts.^14^ This study is included on the Clinical Research Network Portfolio through which the study will be advertised to practices in their regions. Participants will be purposively sampled to allow for maximal variation of healthcare practices according to regional location and local levels of deprivation and according to participants’ years practicing and professional standing as practice, advance, or specialist HF nurse and partner or salaried GP. Interested participants will contact the study team to arrange an interview in-person, by telephone, or video call. Informed consent will be audio recorded at the interview.

#### Data collection

Topic guides have been developed and piloted with input from a GP, nurse, Patient and Public Involvement (PPI) group, charity group, and GP champion from the Clinical Research Network (CRN). One-to-one, semi-structured interviews, following the topic guide will explore GPs’ and nurses’ perspectives on their experiences of consultations with PWHF involving physical activity discussions and any barriers or facilitators to advising and referring to supportive services. The following will be explored: attitudes and perceptions about discussing physical activity with PWHF, confidence and understanding, physical activity resources, and access for PWHF. Interviews will be audio-recorded, transcribed verbatim, anonymised, and managed in NVivo (version 13). Interviews will be done by JP and are estimated to be 30–45 minutes in duration.

Participants will be offered a £50 shopping voucher.

#### Analysis

An exploratory inductive approach to analysis will be adopted to identify the main themes and patterns. An inductive approach identifies themes from the data using a coding process that does not try to fit the data into a pre-existing coding frame or align with the researcher’s analytic preconceptions. This process will mean insights gleaned from previous interviews could inform the next, and codes and themes will be continually developed and refined. JP and RO will both conduct the primary analysis and be responsible for undertaking all coding and theme formulation. They will work with our PPI and co-investigator group to verify initial coding and to discuss emerging themes.

This process, involving PPI and, if appropriate, members of the core research team, will create continual opportunity for the interpretations of the primary analysis to be challenged and refined, thereby enhancing confirmability and rigour. Our PPI group will be supported to read transcribed text and identify key themes and issues from their perspective. This will ensure that the issues relevant to patients are kept central to the analysis.

### Profiling study

#### Aim and design

The aim is to identify what physical activity services are available in England to PWHF and how community healthcare professionals can refer patients to these services.

This will be a scoping study of community physical activity services that are available to PWHF in England.

Information about physical activity services that are available to PWHF in England will be obtained through websites and contacting the Integrated Care Boards in England and those who organise, deliver, and run community physical activity services (for example, gym trainers, programme managers, council staff, NHS staff, charity workers). The method may develop iteratively through the project depending on sources of data available due to the variability in service providers of physical activity for PWHF. We will devise a set of search terms with the aim of identifying community services that support physical activity to all PWHF. We will identify both services that healthcare professionals (GPs and nurses) can refer patients to and, if possible, services that are indirectly referred to via link workers or social prescribers based in primary and community care. We will also obtain data from the National Audit of Cardiac Rehabilitation (cardiac-rehabilitation.net). Correspondence with some individuals may lead to alternative sources from which to obtain further information. Our provisional plan is to collect data on the number of people being referred, uptake, and any measure of effectiveness, for example participation satisfaction. These data may be available through evaluation information via Freedom of Information requests, to councils, for example. We will also consider contacting these services individually if feasible.

#### Sampling

##### Data collection

The data we aim to collect will include what types of provision are available, how they are funded, where they are delivered, who delivers them, and with what frequency. Where this information is not presented on websites, we will request the information from the relevant contact details listed. The sources of information, along with data of interest will be entered into excel spreadsheets. A summary of provisions found for each Integrated Care Board (ICB) region will be presented in PowerPoint.

##### Analysis

Analysis will include both narrative description and summary statistics of the collected data as described above of the provisions accessible to PWHF across England. A visual overview of types of provisions that PWHF can access in England will be made in PowerPoint, with the data presented in summary tables and graphically.

It is possible that some areas may have no provision, but equally we may identify services with detailed information or evaluation material. We will also look at the spread of data across England to consider how it may vary in areas of deprivation. We will follow a cycle of iterative refinement and learning acknowledging contextual variation and being responsive to those contexts by adopting the best-fit analytical approaches that is congruent with type and nature of data in those contexts.

These data will be shared with our public contributors. We will invite them to give their interpretation of the findings to check that we are addressing the issues that are of most relevance to them. Our overall summary of this work will allow us to produce a typology of services.

### Stakeholder engagement

Two workshops will be held to discuss the implications of the findings from the interviews and profiling of community physical activity services. The workshops will be online and we will plan for attendance of healthcare professionals (for example GPs and nurses), specialist exercise instructors, ICB colleagues, charity representatives, Health Integration Team (Active Lives), and 2–3 members of our PPI group. The findings from the interviews will be presented in the first workshop to consider how this information can be shared in practical, plain English summaries for the public and for healthcare professionals. The data from the profiling study will be discussed in the second workshop in terms of how this information should be written up and how it relates to workstream one (qualitative interviews) content. In both workshops, we will discuss how the findings from the two studies inform our initial logic model for supporting physical activity for PWHF, which to date is based mostly on evidence from patient experience.

### Patient and public involvement

A PPI group of 5 members with lived experience of HF have contributed to the development of this research. We will hold PPI meetings and involve our PPI contributors in the workshops. These meetings will seek their input on key stages of the project in relation to the topic guide for the interviews and the content of the questions to be used to profile the community physical activity services. Discussing the developing analysis and findings of the qualitative interviews with our PPI group will also help ensure that their perspective is kept central in the analysis. We will also discuss the data collected on commissioned community physical activity services with our PPI members, asking them how it relates to their experiences and what has helped or hindered them in any participation of similar services. Our PPI’s role in the final stage of write up and the workshops will be essential to ensure that all outputs are patient focused and relevant. We will co-create a plain English summary of our findings with our PPI advisors so that the information can be easily understood by service users and providers.

## Discussion

This study will help address the evidence gap in respect of community healthcare professional conversations about physical exercise with PWHF, professionals’ potential to refer on appropriately, and the referral pathways and community support networks available to PWHF regarding physical activity. The study will also include workshop discussions with community healthcare professionals, during which findings from the study will be shared and debated. Data arising from the study will inform practical recommendations for healthcare professionals, patients, and their support networks.

## Data

Any data not in the published article will be available from the authors on reasonable request.
